# Circular RNA circ_0061140 accelerates hypoxia-induced glycolysis, migration, and invasion in lung adenocarcinoma through the microRNA-653/hexokinase 2 (HK2) axis

**DOI:** 10.1080/21655979.2021.2000743

**Published:** 2022-04-12

**Authors:** Shaobin Wang, Hao Zhang, Lixia Xia, Fen Lan

**Affiliations:** Department of Respiratory and Critical Care Medicine, Second Affiliated Hospital of Zhejiang University School of Medicine, Hangzhou, China

**Keywords:** Lung adenocarcinoma, circ_0061140, miR-653, HK2

## Abstract

Circular RNA (circRNA) is considered to be an essential regulator of multiple human malignancies. However, the role and molecular mechanism of circ_0061140 in lung adenocarcinoma ((LUAD) remain elusive. The levels of circ_0061140, microRNA (miR)-653 and hexokinase 2 (HK2) were examined by RT-qPCR. Downstream targets of circ_0061140 were predicted by circinteractome website and verified by luciferase reporter and RIP assays. HK2 protein level was assessed via Western blotting. The migratory and invasive abilities of LUAD cells were assessed via wound healing and transwell assays. It was uncovered that circ_0061140 level was elevated in LUAD samples, and the high level of circ_0061140 was related to poor survival rate of LUAD patients. Circ_0061140 deletion inhibited glycolysis, migration and invasion of hypoxia-treated LUAD cells. Moreover, circ_0061140 could modulate HK2 level by absorbing miR-653. Furthermore, miR-653 silence or HK2 addition neutralized the effects of circ_0061140 knockdown on LUAD progression under hypoxia. This study elaborated that circ_0061140 accelerated hypoxia-triggered glycolysis, migration and invasion in LUAD cells via downregulating miR-653 and increasing HK2 expression.

## Introduction

Lung cancer is one of the most common malignancies and has the highest mortality rate in the world [1]. Lung adenocarcinoma (LUAD), a major subclass of lung cancer, accounts for nearly 40% of lung cancer cases [2]. In the past few decades, the incidence of LUAD has increased significantly, posing a major threat to human life and health [3,4]. Despite extensive efforts have been paid in the diagnosis and treatment of LUAD, it remains the most aggressive and fastest fatal disease, with a 5-year survival rate < 15% [5]. Thus, the identification of new molecular markers is important for the early diagnosis and treatment of LUAD.

Circular RNAs (circRNAs) are a class of non-coding RNAs (ncRNAs) that harbor covalently closed-loop structures [6]. Recently, it attracted more attention about circRNAs’ function in the progression of malignant tumors, including LUAD. For example, circ_0027446 aggregated LUAD cell metastasis via regulating miR-1236-3p and increasing ZEB1 [7]. CircPRKCI facilitated cell viability and migration in LUAD via the miR-219a-5p/CAMK1D axis [8]. CircPUM1 accelerated the tumorigenesis of LUAD through interacting with miR-326 [9]. Circ_0061140, a newly discovered circRNA, has been shown to act as a carcinogenic function in several cancer types. For instance, circ_0061140 expedited cell viability and invasion by modulation of miR-1236 in bladder cancer [10]. Besides, circ_0061140 aggravated prostate cancer progression through absorbing miR-1193 [11]. Nonetheless, the mechanism of circ_0061140 in LUAD development remains elusive.

MicroRNAs (MiRNAs) are a group of non-coding RNAs containing ~ 22 nts, with a role in regulating gene expression [12]. Dysregulated miRNAs were reported to participate in LUAD progression. Wang et al implied that miR-335-5p modulated the downregulation of CCNB2 to suppress LUAD development [13]. Li et al implied that miR-490-3p repressed LUAD cell viability and accelerated apoptosis through inactivating the Wnt/β-catenin signaling [14]. Tong et al displayed that miR-365 attenuated LUAD cell viability, migration, and invasion via regulating ETS1 and inactivating AKT/mTOR pathway [15]. miR-653, a miRNA related to tumorigenesis, has been confirmed to be a tumor suppressor in various cancers, such as melanoma and breast cancer [16,17]. Besides, hexokinase 2 (HK2) is an essential enzyme associated with glucose metabolism and is necessary for glycolysis, proliferation, and migration in lung cancer [18]. Nevertheless, whether miR-653 and HK2 participated in the circ_0061140-mediated mechanism in LUAD remains obscure.

This work aimed to explore the expression and underlying mechanism of circ_0061140 in LUAD. It was hypothesized that circ_0061140 was upregulated in LUAD and promoted migration, invasion, and glycolysis in hypoxia-induced LUAD cells via the miR-653/HK2 axis.

## Materials and methods

### Tissues samples

38 pairs of LUAD tissues and matched normal lung tissues were acquired from the Second Affiliated Hospital of Zhejiang University School of Medicine. All specimens were immediately conserved at −80°C for use. Patients did not receive systemic treatment of chemotherapy or radiotherapy before surgery and written informed consents from patients were gained. This study was permitted by the Ethics Committee of the Second Affiliated Hospital of Zhejiang University School of Medicine.

### Cell culture and hypoxia treatment

Human LAUD cell lines (A549, NCI-H23, H1975, and H1299) and a normal human bronchial epithelial cell line (HBE) were gained from ATCC (Manassas, Virginia) and cultured in RPMI-1640 medium (Invitrogen) with 10% (FBS under 5% CO_2_ at 37°C. For hypoxia stimulation, A549 and NCI-H23 cells were grown in a hypoxia chamber with 1% O_2_ for different time points (0, 6, 12, 24, and 48 h).

### Cell transfection

Small interfering RNA (siRNA) against circ_0061140 (si-circ_0061140), negative control (si-NC), miR-653 mimics, NC mimics, miR-653 inhibitor and NC inhibitor were purchased from Ribobio (Guangzhou, China). HK2 sequence was cloned into pcDNA3.1 to construct pcDNA3.1/HK2. The transfection was done with Lipofectamine 2000 (Invitrogen).

### Reverse transcription-quantitative PCR (RT-qPCR) assay

Total RNAs were extracted using TRIzol Kit (Invitrogen). RNA was reverse-transcribed into cDNA using a PrimeScript RT reagent Kit (Takara). Then, RT-qPCR was conducted using SYBR-Green PCR kit (Applied Biosystems). The 2^–ΔΔCT^ method was used for the calculation of gene expression. GAPDH or U6 were regarded as controls [19].

### Wound healing assay

Briefly, A549 and NCI-H23 cells were seeded in 6-well plates. Then, the scratches were made with a pipette tip. Cells were starved and cultured for 24 h. Cell migration was analyzed using an inverted microscope (Olympus Corporation) [20].

### Examination of glucose consumption and lactate production

A549 and NCI-H23 cells were added into 24-well plates and stimulated by hypoxia for 24 h. Glucose consumption and lactate production were examined using a Glucose Uptake Colorimetric Assay Kit and Lactate Colorimetric Assay Kit (Biovision, San Francisco, CA) [21].

### Western blot

Total protein was isolated using RIPA buffer (Beyotime), exposed to 10% SDS-PAGE, and transferred onto PVDF membranes (Millipore). Next, the membranes were blocked in 5% skim milk, and then interacted with primary antibodies containing anti-HK2 (ab209847, 1:500, Abcam), and anti-GAPDH overnight at 4 C. Following the incubation of secondary antibody for 1 h. The protein signals were analyzed by the ECL system kit (Pierce, Rockford, USA) [22].

### Transwell invasion assay

The invasive ability was detected via transwell invasion assay using transwell chambers (pore size 8 μm) coated with Matrigel (Sigma). Briefly, transfected cells were seeded into the apical chamber with free-serum medium. The bottom chambers were filled with complete RPMI-1640 medium. After 24 h, cells in the bottom chamber were dyed with crystal violet (Sangon). Cell numbers were counted by a microscope (Nikon, Japan) [23].

### Dual‑luciferase reporter assay

The sequences of circ_0061140 and HK2 3’-UTR containing wild-type or mutated miR-653 binding sites were cloned into pmirGLO reporter vectors (Promega), namely circ_0061140-WT, circ_0061140-Mut, HK2-WT, or HK2-Mut. The miR-653 mimics or NC mimics and the above vectors were co-transfected into cells. After 48 h, the luciferase activity was detected using the dual-luciferase detection system (Promega).

### RNA immunoprecipitation (RIP) assay

RIP assay was performed using the EZ-Magna RIP kit (Millipore). A549 and NCI-H23 cells transfected with miR-653 mimics or NC mimics were lysed in RIP lysis buffer, cell lysates were treated with magnetic beads combined with anti-Ago2 or anti-IgG. The abundances of circ_0061140, miR-653, and HK2 were measured by RT-qPCR [24].

### Statistical analysis

Data analyses were assessed using SPSS 21.0 (IBM) and exhibited as the mean ± SD. The comparison was performed with Student’s t-test or one-way ANOVA. Survival curves were generated by Kaplan-Meier method. Pearson correlation analysis assessed the interaction between genes. P < 0.05 had statistical significance.

## Results

In this study, we investigated the biological role and molecular mechanism of circ_0061140 in LAUD. Functional assays revealed that knockdown of circ_0061140 inhibited hypoxia-induced glycolysis, migration, and invasion in LAUD via sponging miR-653 and modulating HK2 expression.

### Circ_0061140 is upregulated in LAUD under hypoxia

**Figure 1.**
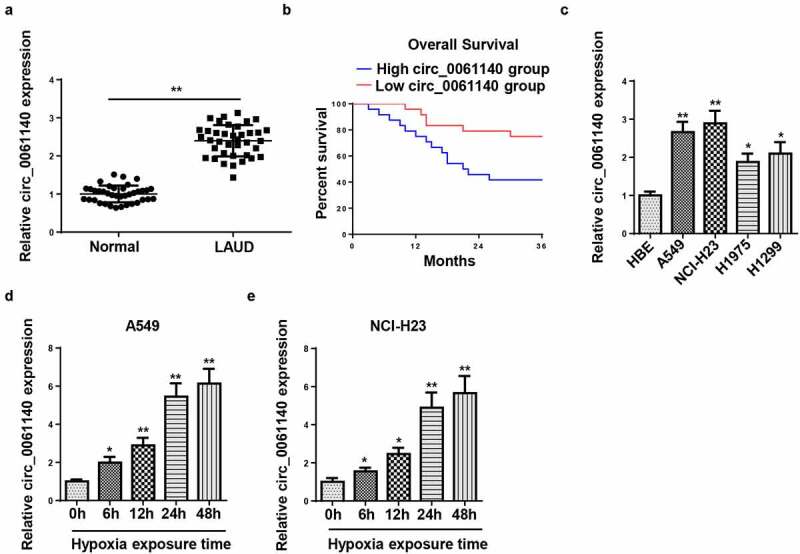
Circ_0061140 is upregulated in LAUD under hypoxia. (a) RT-qPCR was used to detect the expression of circ_0061140 in LAUD tissues. (b) Kaplan-Meier method performed the survival curves of circ_0061140 in LAUD patients. (c) RT-qPCR showed the expression of circ_0061140 in OC cell lines (A549, NCI-H23, H1975 and H1299) and human bronchial epithelial cell line (HBE). (d and e) RT-qPCR showed circ_0061140 expression in A549 and NCI-H23 cells after 1% hypoxia exposure for 0, 6, 12, 24, and 48 h. *P < 0.05, **P < 0.01.

Firstly, we detected the abundance of circ_0061140 in LAUD tissues by RT-qPCR. As exhibited in Figure 1a, circ_0061140 level was enhanced in LAUD tissues (Figure 1a), and the high level of circ_0061140 was related to the low survival rate of LAUD patients (Figure 1b). Moreover, RT-qPCR results displayed that circ_0061140 level was elevated in LAUD cell lines (Figure 1c). A549 and NCI-H23 cells were chosen for further experiments due to the highest circ_0061140 expression. Besides, the level of circ_0061140 was tested in LAUD cells after exposure to hypoxia. Results exhibited that the abundance of circ_0061140 was enhanced in A549 and NCI-H23 cells after hypoxia treatment in a time-dependent manner (Figure 1d and e). These data manifested that circ_0061140 was upregulated in LAUD cells under hypoxia.

### Circ_0061140 deletion hampers hypoxia-stimulated glycolysis and metastasis of LAUD cells

**Figure 2.**
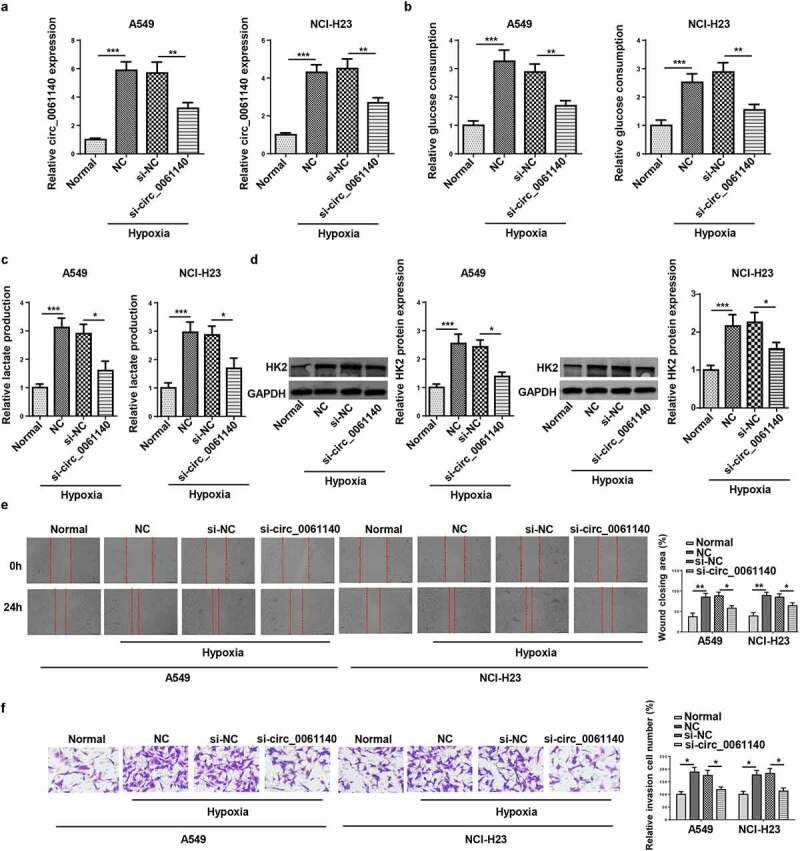
Circ_0061140 deletion hampers hypoxia-stimulated glycolysis and metastasis of LAUD cells. (a) RT-qPCR showed the expression of circ_0061140 in A549 and NCI-H23 cells transfected with si_circ_0061140 or si-NC in LAUD under hypoxic condition. (b-d) Glycolysis was evaluated by the glucose consumption, lactate production, and Western blotting showed glycolysis-associated enzyme HK2 protein level. (e and f) Wound healing and transwell assays showed the migration and invasion of A549 and NCI-H23 cells transfected with si_circ_0061140 or si-NC in LAUD under hypoxic condition. *P < 0.05, **P < 0.01, ***P < 0.001.

Then, the regulatory role of circ_0061140 in LAUD progression under hypoxia was explored. It was manifested that circ_0061140 level was decreased by circ_0061140 deletion in hypoxia-treated LAUD cells (Figure 2a). Moreover, hypoxia treatment increased glucose consumption and lactate production in A549 and NCI-H23 cells, while circ_0061140 silence abrogated these effects (Figure 2b-c). Furthermore, Western blotting determined that HK2 protein level was elevated by hypoxia treatment, which was decreased via circ_0061140 deficiency (Figure 2d). Besides, LAUD cell migration and invasion were promoted by hypoxic stimulation, while circ_0061140 interference mitigated these effects (Figure 2e and f). The above data elaborated that circ_0061140 knockdown suppressed LAUD development under hypoxia.

### miR-653 is sponged by circ_0061140

**Figure 3.**
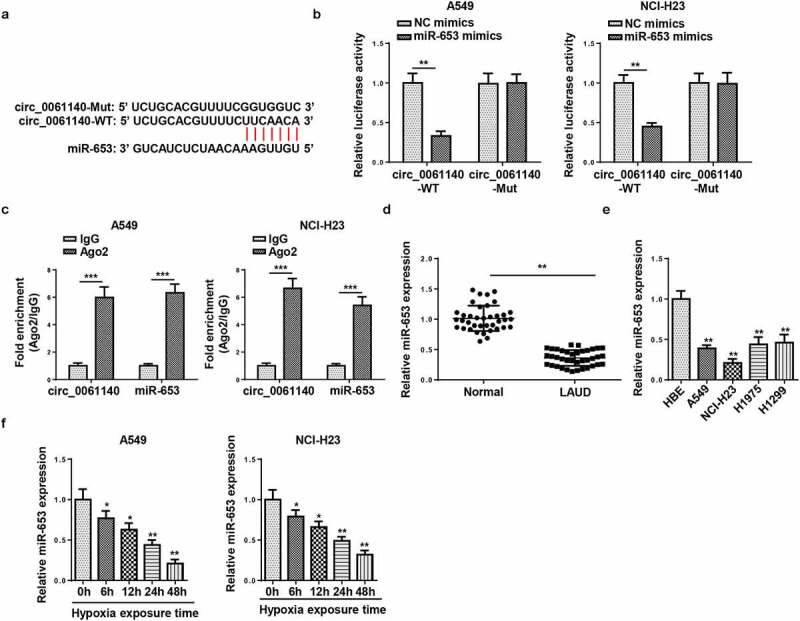
miR-653 is sponged by circ_0061140. (a) Putative binding regions of circ_0061140 in miR-653 predicted with Circular RNA Interactome. (b) Luciferase reporter assay showed the luciferase activity of wild-type or mutant circ_0061140 in A549 and NCI-H23 cells transfected with NC mimics or miR-653 mimics. (c) RIP assay showed the levels of circ_0061140 and miR-653 of A549 and NCI-H23 cells in Ago2 group. (d and e) RT-qPCR analysis was used to detect the expression of miR-653 in LAUD tissues and cells. (f and g) The abundances of miR-653 was detected in A549 and NCI-H23 cells after exposure of hypoxia. The data were presented as mean ± SD *P < 0.05, **P < 0.01.

The downstream regulatory mechanism of circ_0061140 in LAUD was elucidated, and the targets of circ_0061140 were predicted by Circular RNA Interactome (https://circinteractome.nia.nih.gov/). The binding sites of circ_0061140 and miR-653 were displayed in Figure 3a. To confirm this interaction, luciferase reporter and RIP assays were conducted in A549 and NCI-H23 cells. Results determined that the addition of miR-653 restrained the activity in circ_0061140-WT group, but not in circ_0061140-Mut group (Figure 3b). Moreover, high enrichment of circ_0061140 and miR-653 was identified in Ago2 group (Figure 3c). Furthermore, RT-qPCR results displayed that miR-653 level was reduced in LAUD tissues and cells (Figure 3d and e). Also, miR-653 was downregulated by hypoxia treatment in LAUD cells (Figure 3f and g). Overall, we concluded that circ_0061140 targeted miR-653 in LAUD cells.

### HK2 is targeted by miR-653

**Figure 4.**
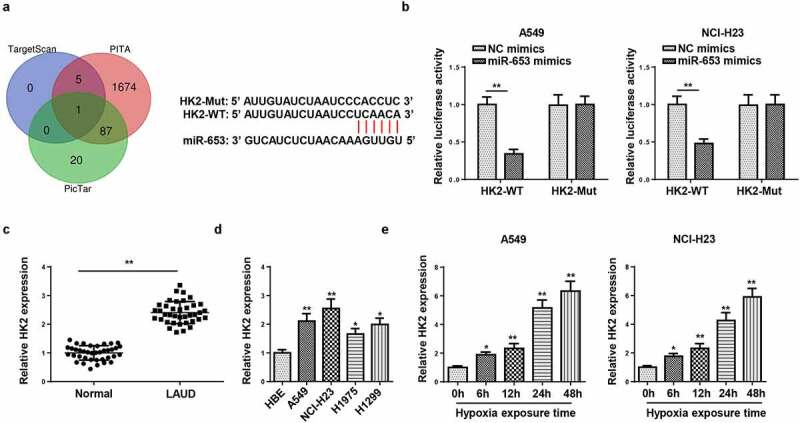
HK2 is targeted by miR-653. (a) Venn diagram analysis showed the potential target of miR-653. (b) Luciferase reporter gene assay showed the luciferase activity of wild-type or mutant HK2 in A549 and NCI-H23 cells transfected with NC mimics or miR-653 mimics. (c and d) RT-qPCR analysis showed the expression of HK2 in LAUD tissues and cell lines. (e) RT-qPCR determined HK2 expression in A549 and NCI-H23 cells after hypoxia exposure for 0, 6, 12, 24, and 48 h. *P < 0.05, **P < 0.01.

Subsequently, the target genes that may bind to miR-653 were predicted through bioinformatics websites (TargetScan, PITA, and PicTar), and we uncovered that HK2 was a potential target of miR-653 (Figure 4a). Moreover, it was confirmed that the luciferase activity in HK2-WT was inhibited by miR-653 supplementation, but no evident changes in HK2-Mut group (Figure 4b). Moreover, HK2 level was augmented in LAUD tissues and cells (Figure 4c and d). Similarly, RT-qPCR determined that HK2 expression was elevated by hypoxia stimulation in A549 and NCI-H23 cells (Figure 4e). Therefore, HK2 was directly targeted by miR-653.

### Circ_0061140 modulates HK2 expression through absorbing miR-653

**Figure 5.**
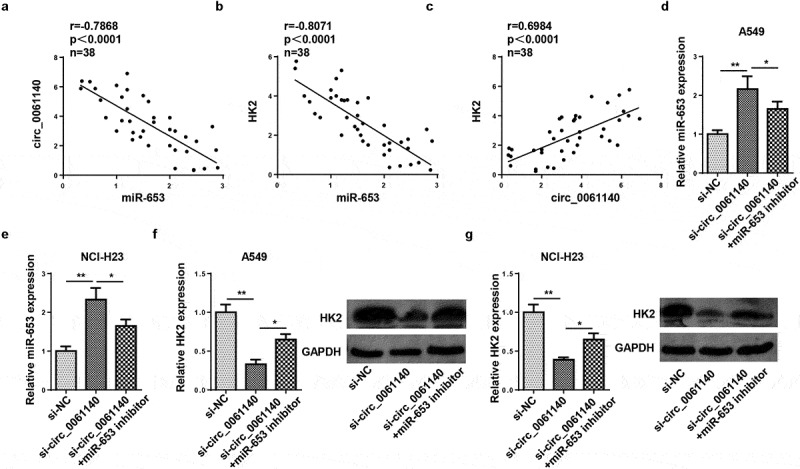
Circ_0061140 modulates HK2 expression through absorbing miR-653. (a and b) Pearson correlation analysis showed the correlation between miR-653 and circ_0061140 or HK2 expression in LAUD tissues. (c) Pearson correlation analysis showed the correlation between circ_0061140 and HK2 expression in LAUD tissues. (d and e) RT-qPCR analysis showed the expression of miR-653 in A549 and NCI-H23 cells transfected with si-NC, si_circ_0061140 and si_circ_0061140+ miR-653 inhibitor. (f and g) Western blotting analysis showed the expression of HK2 in A549 and NCI-H23 cells transfected with si-NC, si-circ_0061140 and si-circ_0061140+ miR-653 inhibitor. *P < 0.05, **P < 0.01.

To study whether circ_0061140 modulated HK2 level, the relationship among circ_0061140, miR-653, and HK2 was assessed. We identified that miR-653 level was inversely correlated with circ_0061140 or HK2 (Figure 5a and b). However, the HK2 levels were positively related to circ_0061140 levels (Figure 5c). Next, RT-qPCR analysis displayed that miR-653 level was enhanced by circ_0061140 knockdown, which was weakened by miR-653 inhibition in A549 cells (Figure 5d and e). Meanwhile, HK2 protein level was decreased by circ_0061140 knockdown, which was reversed by miR-653 inhibtion (figure 5f and g). In sum, we concluded that circ_0061140 positively modulated HK2 by targeting miR-653 in LAUD cells.

### Circ_0061140 deficiency represses LAUD progression by regulating miR-653 and HK2 under hypoxia

**Figure 6.**
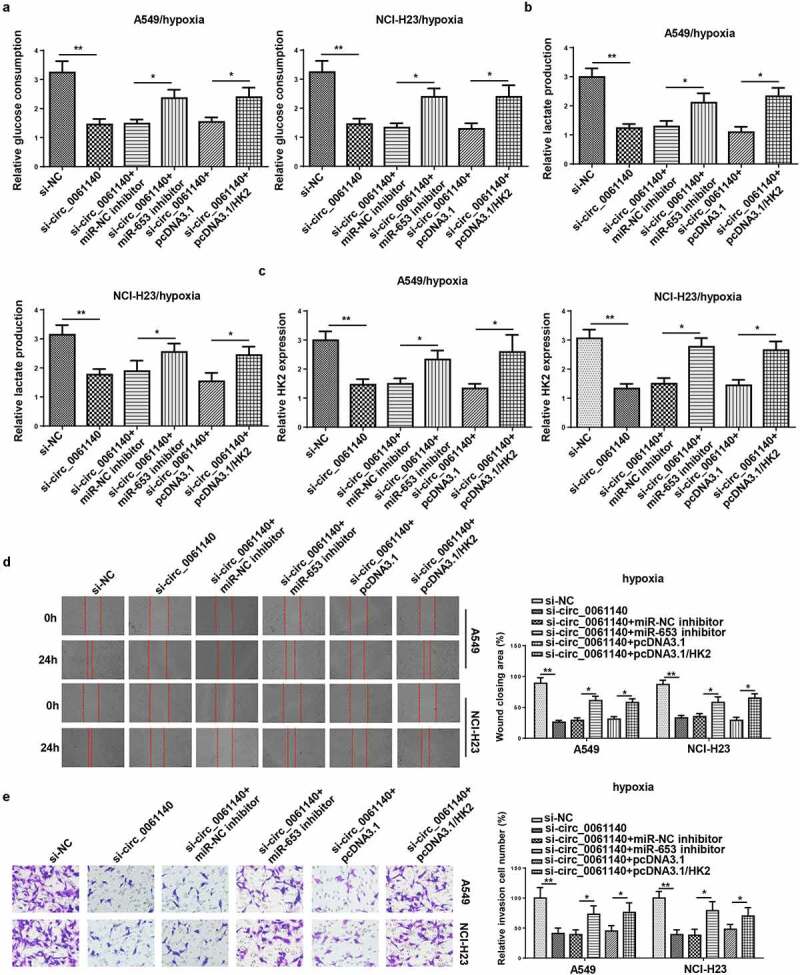
Circ_0061140 deficiency represses LAUD progression by regulating miR-653 and HK2 under hypoxia. (a–c) Glycolysis was evaluated by the glucose consumption, lactate production, and RT-qPCR showed HK2 expression in A549 and NCI-H23 cells transfected with si-NC, si-circ_0061140, si-circ_0061140+ miR-NC inhibitor, si_circ_0061140+ miR-653 inhibitor, si-circ_0061140+ pcDNA3.1, and si-circ_0061140 + pcDNA3.1/HK2. (d and e) Wound healing and transwell assays showed the migration and invasion of A549 and NCI-H23 cells transfected with si-NC, si-circ_0061140, si-circ_0061140+ miR-NC inhibitor, si-circ_0061140+ miR-653 inhibitor, si-circ_0061140+ pcDNA3.1 and si-circ_0061140 + pcDNA3.1/HK2. The data were presented as mean ± SD *P < 0.05, **P < 0.01.

To investigate whether miR-653 and HK2 participated in circ_0061140-mediated glycolysis, migration, and invasion of LAUD under hypoxia, A549 and NCI-H23 cells were transfected with si-NC, si-circ_0061140, si-circ_0061140+ miR-NC inhibitor, si-circ_0061140+ miR-653 inhibitor, si-circ_0061140+ pcDNA3.1 and si-circ_0061140+ pcDNA3.1/HK2. We discovered that miR-653 silence or HK2 supplementation rescued the suppressive effects of circ_0061140 silence on glucose consumption, lactate production, HK2 expression in A549 and NCI-H23 cells under hypoxia (Figure 6a–c). Meanwhile, circ_0061140 deficiency suppressed the migration and invasion of LAUD cells under hypoxia, while miR-653 knockdown or HK2 overexpression abated these effects (Figure 6d and e). Taken together, circ_0061140 deficiency hindered LAUD development under hypoxia through regulating the miR-653/HK2 axis.

## Discussion

Accumulating studies illuminated the crucial role of circRNAs in the biological processes of diverse malignancies. For example, deficiency of circ_0013958 restrained cell viability and metastasis, but facilitated cell apoptotic in ovarian cancer [25]. circFBLIM1 functioned as a ceRNA to expedite the viability and invasion of hepatocellular cancer cells via mediating miR-346 [26]. Circ_0061140 has been reported as an oncogene in serval human cancers, such as prostate cancer [11], bladder cancer [10], endometrial cancer [27]. In addition, intratumoral hypoxia has been identified as a driving force of tumor progression [28,29], and circRNA could be regulated by hypoxia in tumors [30,31]. In this work, we identified that circ_0061140 level was alerted by hypoxia stimulation, suggesting that circ_0061140 might act as a crucial function in LAUD development under hypoxia. In addition, functional assays revealed that the silencing of circ_0061140 attenuated hypoxia-stimulated glycolysis, migration, and invasion in LAUD.

CircRNAs exhibited their biological roles by acting as ceRNAs of miRNAs in human cancers [32]. It was reported that circ_0061140 modulated tumor development by sponging miRNAs. For example, circ_0061140 enhanced the viability rate and invasion of bladder cancer cells via absorbing miR-1236 [33]. Circ_0061140 interference inhibited cell growth and metastasis via regulating the miR-370/FOXM1 axis in ovarian cancer [34]. Here, we confirmed that circ_0061140 could target miR-653. miR-653 was implied to be an anti-tumor gene in some cancers. For example, lncRNA PLK1S1 expedited renal cell carcinoma cell viability and invasion by absorbing miR‑653 and altering CXCR5 level [35]. Moreover, circHIPK3 facilitated gastric cancer metastasis through interacting with miR-653 and miR-338-3p to modulate NRP1 expression under hypoxia [36]. This research implied that miR-653 level was reduced in LAUD cells treated by hypoxia. miR-653 deletion reversed circ_0061140 silence‑induced effects on the glycolysis and metastasis of hypoxia‑stimulated LAUD cells.

It is well known that the biological effects of miRNAs are achieved by regulating mRNA level, so we used bioinformatics websites to predict the potential target gene of miR-653, HK2 was selected as the target of miR-653 because of its tumor-promoting impact. Previous studies have shown that HK2 is essential for glucose metabolism in various cancers. Wang et al exhibited that miR-202 impaired pancreatic cancer glycolysis and repressed cell proliferation by increasing HK2 [32]. Besides, Liu et al implied that HK2 was highly expressed in breast cancer, and HK2 supplementation reversed the suppressive impacts of miR-143-3p addition on breast cancer cell behaviors [33]. Herein, we uncovered that HK2 was overexpressed in LAUD tissues and cells, and HK2 addition neutralized the influence of circ_0061140 inhibition on LAUD progression. These results elucidated that circ_0061140 accelerated hypoxia-driven LAUD development via the miR-653/HK2 axis.

## Conclusion

Our study demonstrated that circ_0061140 was upregulated in LAUD under hypoxia. Moreover, circ_0061140 deficiency mitigated hypoxia-induced glycolysis, migration and invasion in LAUD via sponging miR-653 and modulating HK2, suggesting that circ_0061140 might be a novel therapeutic and diagnostic target for LAUD.

## Data Availability

The datasets generated during and/or analyzed during the current study are available from the corresponding author on reasonable request.
